# An Evaluation Method for Partial Discharge in Generator Stator Bar Insulation Based on Fiber-Optic Acoustic Detection

**DOI:** 10.3390/s26072053

**Published:** 2026-03-25

**Authors:** Jianlin Hu, Jiapeng Yang, Peiyu Qin, Xingliang Jiang, Wentao Luo

**Affiliations:** School of Electrical Engineering, Chongqing University, Chongqing 400044, China; 202311021172t@stu.cqu.edu.cn (J.Y.); 202211021108t@cqu.edu.cn (P.Q.); xljiang@cqu.edu.cn (X.J.); 202411131297@stu.cqu.edu.cn (W.L.)

**Keywords:** partial discharge, fiber-optic acoustic detection, generator stator bar, long time-series modeling

## Abstract

Partial-discharge (PD) monitoring is essential for assessing the insulation condition of generator stator bars. Conventional methods are susceptible to electromagnetic interference and are difficult to deploy in confined stator geometries. Fiber-optic acoustic detection technology offers strong immunity to electromagnetic interference and is suitable for the narrow and high-interference environment of stator bars, but it cannot directly provide discharge magnitude information. Therefore, in this study, fiber-optic acoustic detection technology was employed to acquire partial discharge acoustic signals from stator bars, and a mandrel-type fiber-optic acoustic sensor was developed, with PD tests performed on full-scale stator bars with internal defects. Meanwhile, considering the complex temporal characteristics of PD acoustic signals, a hybrid neural network—Transformer–convolutional neural network–long short-term memory (Transformer–CNN–LSTM)—was constructed for long-term time-series modeling to establish the mapping between acoustic signals and discharge magnitude intervals. The results indicate that fiber-optic acoustic detection enables sensitive and stable detection of weak PD acoustic signals. Phase-resolved PD (PRPD) patterns from the proposed system align with the discharge characteristics of internal defects, with the acoustic signal showing a phase lag relative to the electrical PD signal. The hybrid model achieved an overall interval estimation accuracy of 96.6%, outperforming CNN and CNN-LSTM models, with accuracies of 100% and 99.4% for discharge magnitude intervals below 100 pC and above 2000 pC, respectively.

## 1. Introduction

Partial-discharge (PD) detection is an effective method for evaluating insulation degradation in generators and other power equipment [1]. Monitoring PD activity enables the early identification of hidden defects and timely fault warning [2]. In the field of PD detection, electrical detection methods, such as the pulse-current method and the ultra-high-frequency (UHF) method, have demonstrated high sensitivity but pose electromagnetic compatibility issues [3,4]. Non-electrical techniques, by contrast, identify secondary PD effects, including acoustic, optical, and thermal emissions. Traditional acoustic testing typically employs a piezoelectric transducer (PZT) in a stethoscope-like manner, making it susceptible to electromagnetic interference, while cable attenuation restricts the signal transmission distance [5]. Zheng et al. applied the pulse-current method at cable terminations to detect PD signals and examined their attenuation along the line, but they overlooked the distortion caused by the excessive loss of high-frequency components [6]. Sun et al. proposed a UHF-based hybrid detection approach to analyze insulation PD in more-electric aircraft motors under low pressure; however, their experiments were limited to square-wave voltage excitation [7]. Wang et al. developed an interferometric acoustic sensor with a vibration-coupled air gap that detected acoustic PD signals from stator slots; however, its narrow 8 kHz bandwidth limited coverage of other spectral components [8].

Fiber-optic acoustic sensing provides high sensitivity, long service life, compact size, strong resistance to electromagnetic interference, and flexible installation [9]. The complex electromagnetic environment and limited space at the generator end-windings make fiber-optic acoustic sensing a promising technique for detecting partial discharge in stator bars [10]. Phase-sensitive OTDR (Φ-OTDR) detects extremely weak vibrations in optical fibers by capturing highly sensitive phase variations, providing higher detection accuracy and broader application prospects in the field of fiber-optic sensing [11]. Recent studies have examined its application to various power apparatus. For power cables, Qin et al. applied dual-frequency probe pulses in Φ-OTDR to mitigate periodic vibrations and fading noise in cable PD monitoring, but the approach has shortcomings such as low spatial resolution and limited bandwidth [12]. In GIS applications, Chen et al. integrated a 3D-printed elastomer with Φ-OTDR, enhancing the phase peak-to-peak response from 0.8 to 4 rad; however, the need for phase differencing to offset acousto-optic frequency drift reduced system stability [13]. For transformers, Kirkcaldy et al. at the University of Southampton employed OTDR under undersampling conditions to detect 223 pC PD at the oil-pressboard interface; however, the 20 kHz sampling rate risked missing higher-frequency components, restricting the effective detection bandwidth [14].

Although advances have been made for cables, GIS, and transformers, studies targeting PD in generator stator bars remain limited, with insufficient evidence regarding the acoustic response of internal-defect discharges [15]. Currently, related studies focus mainly on discharge identification and localization, and relatively few have investigated the mapping relationship between acoustic signals and partial discharge magnitude. Most studies establish empirical models based on statistical features such as amplitude or energy, resulting in limited quantitative capability [16,17]. Meanwhile, deep learning enables the automatic extraction of complex temporal features and the construction of nonlinear mapping relationships, making it particularly suitable for analyzing long-term nonstationary acoustic signals [18].

Therefore, this study introduces a Rayleigh-backscattering (RBS)-based fiber-optic acoustic detection method for PD detection in generator stator bars. A fiber-optic acoustic sensor with enhanced sensitivity and stability was developed, and its interference resistance was evaluated. Validation was carried out through experiments on full-scale stator bars, integrating the pulse-current method with the proposed fiber-optic acoustic detection technique to verify the feasibility of fiber-optic acoustic detection in stator-bar PD monitoring. In addition, a Transformer–CNN–LSTM hybrid neural network designed for long time series was constructed to establish a mapping between PD acoustic signals and discharge magnitude intervals.

## 2. Fiber-Optic Acoustic Detection of Partial Discharge

### 2.1. Fiber-Optic Sensing System Based on Rayleigh Scattering

The RBS is an elastic process that preserves photon frequency. However, it is highly sensitive to strain and vibration, making it effective for acoustic sensing. When a probe light enters an optical fiber, RBS occurs along its length. Acoustic pressure alters the refractive index and geometry of the fiber through photoelastic and elasto-geometric effects, inducing an optical phase change [19]. The perturbation is quantified by comparing the optical phase before and after the disturbance, with OTDR employed to sample and analyze the RBS signal [20,21].

Assuming that the effective interaction length of the acoustic field acting on the fiber is *L*, the phase delay *φ* induced by acoustic excitation during light propagation in the fiber can be expressed as:

(1) $$\varphi =\beta L=\frac{\omega n}{c}L=nkL$$

In Equation (1), *β* denotes the propagation constant of the optical wave in the fiber, *k* represents the vacuum wavenumber; *ω* is the angular frequency of the optical wave; *n* is the refractive index of the fiber; and *c* denotes the speed of light in vacuum.

Considering the round-trip propagation of the backscattered light, the phase variation can be expressed as:

(2) $$\Delta \varphi =\frac{4\pi n}{\lambda }\;\Delta L$$

In Equation (2), *λ* denotes the wavelength of the incident light, and Δ*L* represents the change in fiber length induced by the acoustic field.

**Figure 1:**
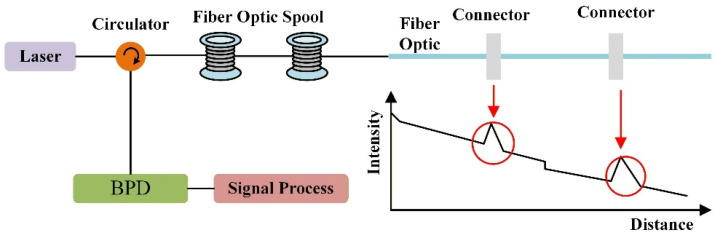
Basic architecture of the OTDR.

Figure 1 illustrates that pulsed light is injected into the sensing fiber through an optical circulator, while the RBS is detected by a photodetector. For a single pulse with round-trip time *T*, each time instant within *T* corresponds to a unique fiber position through time-of-flight mapping. Consequently, the spatial distribution of local disturbances can be reconstructed across the full fiber length.

Coherent detection provides superior sensitivity for weak signals compared with direct detection. Within coherent schemes, heterodyne detection offers a simpler and more economical architecture than homodyne (zero intermediate frequency) schemes while maintaining high sensitivity and phase selectivity. This study employs a coherent heterodyne scheme to detect the acoustic emissions from partial discharge [22].

**Figure 2:**
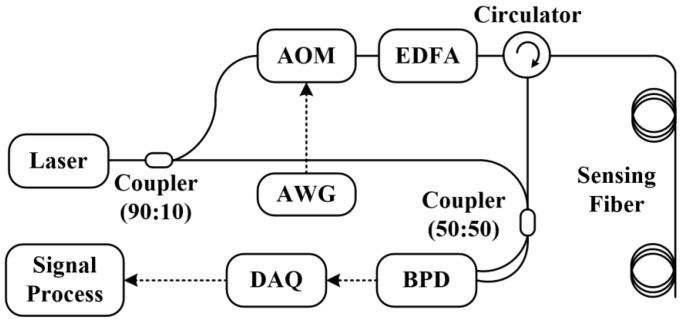
Topology of the fiber-optic sensing system.

As described above, the fiber-optic sensing system is arranged according to the topology illustrated in Figure 2, in which the RBS is acquired and processed to reconstruct the local perturbations along the sensing fiber.

A continuous-wave (CW) laser is divided by a 1 × 2 fiber coupler into a probing arm and a reference arm. In the probing arm, an acousto-optic modulator (AOM), driven by an arbitrary waveform generator (AWG), gates the light into pulses, which are subsequently amplified by an erbium-doped fiber amplifier (EDFA) and launched into the sensing fiber through an optical circulator. The RBS field is routed back through the circulator and heterodyned with the local-oscillator (LO) light from the reference arm at a 2 × 2 coupler. This process generates two interference outputs with a fixed phase relationship. A balanced photodetector (BPD) converts the differential optical input into an electrical waveform, which is digitized by the data-acquisition (DAQ) card. The AWG provides the trigger to synchronize DAQ timing.

The Φ-OTDR system demodulates and acquires the phase variation at each position along the sensing fiber through coherent optical intensity signals [23]. The coherent optical intensity received by the photodetector can be expressed as Equation (3):

(3) $$E={\left|E_{1}\right|}^{2}+{\left|E_{2}\right|}^{2}+2E_{1}E_{2}\cos\left(\theta \right)\cos\left(\Delta \omega t+\varphi \left(t\right)\right)$$

In Equation (3), *E*_1_ and *E*_2_ are the electric field amplitudes of the Rayleigh backscattered light and the local oscillator light, respectively. The parameter *θ* is the polarization angle between the two optical fields at the photodetector. *ω* is the beat angular frequency, and *Φ*(*t*) denotes the optical phase variation induced by the external perturbation. The resulting alternating-current (AC) component *I*(*t*) = 2*E*_1_*E*_2_cos(*θ*)cos(Δ*ωt* + *Φ*(*t*)) can be extracted using the AC-coupling mode of an oscilloscope.

### 2.2. Fiber-Optic Acoustic Sensor Design

The acoustic signals generated by partial discharges in generator stator windings become extremely weak when they propagate to the outer surface of the stator core. In addition, the stator slots of large generators are densely arranged, resulting in very limited installation space. Since partial discharges most frequently occur near the stator slot-exit region, this location provides a certain amount of available space and facilitates the routing and lead-out of the sensing fiber. Therefore, the sensing fiber should be arranged in the region between the stator slot exit and the R-corner of the stator bar.

**Figure 3:**
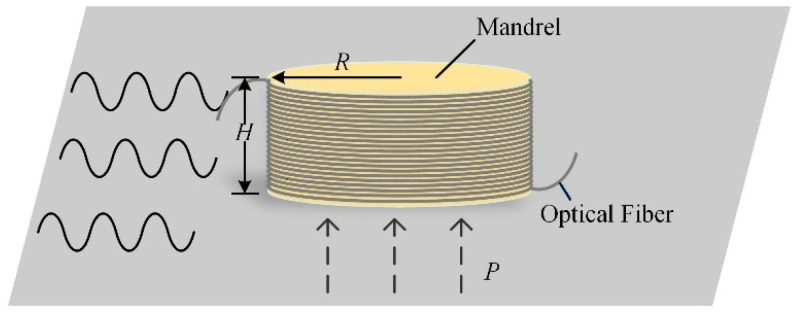
Schematic diagram of sensor structure.

The mandrel-type fiber-optic acoustic sensor helically winds the sensing fiber around a cylindrical mandrel to extend the interaction length between the acoustic field and the fiber and to enlarge the acoustic aperture, as illustrated in Figure 3. This design enhances the detection sensitivity to weak acoustic signals induced by partial discharge and supports conformal attachment to the surface of the stator bar.

In the present fiber-optic sensing architecture, the effective variation in fiber length within a mandrel-type acoustic sensor is mainly governed by mandrel deformation. When an acoustic pressure *P* is applied to the mandrel, an axial stress is generated to balance the external load. For a fiber layer tightly wound on the mandrel, the axial displacement under unit acoustic pressure can be expressed as follows:

(4) $$\Delta r=\frac{\mu HR^{2}}{EHR-\mu E_{f}S_{f}N}P$$

With the fiber layers assumed to be tightly packed and their total thickness negligible compared to the mandrel diameter, the number of fiber turns on the mandrel is approximately given by *N ≈ L/*2*πR*. Combining the relationship between optical wavenumber and wavelength, together with the fiber length dependence of optical phase, the phase variation in the guided light is expressed as follows:

(5) $$\Delta \varphi_{1}=\frac{8\pi^{2}n\mu HR^{2}L}{\lambda (2\pi R^{2}EH-\mu E_{f}S_{f}L)}P$$

where *n* signifies the effective refractive index of the fiber. The frequency dependence of the phase can be expressed as follows:

(6) $$\Delta \varphi_{1}(f)=\Delta \varphi_{1}\cdot \frac{{f_{c\;}}^{2}}{\sqrt{{\left(f^{2}-{f_{c\;}}^{2}\right)}^{2}+{\left(f\cdot f_{c\;}/Q\right)}^{2}}}$$

where *Q* is the damping parameter of the system (typically 1.1–1.2, and *Q* = 1.1 is adopted in this work), and *f_c_* represents the resonant frequency associated with the radial breathing mode of the system.

At radial breathing resonance, the mandrel is treated as a single-degree-of-freedom oscillator, with the resonance frequency defined as follows:

(7) $$f_{c}=\frac{1}{2\pi }\sqrt{\frac{K_{\mathrm{eff}}}{m_{\mathrm{eff}}}}$$

The effective mass is given by the combined contributions of the mandrel and the fiber layer, which is given by:

(8) $$m_{\mathrm{eff}}=\pi \rho_{c}HR^{2}+\pi \rho_{f}R_{f}^{2}L$$

where *ρ_c_* is the density of the mandrel, *ρ_f_* denotes the density of the fiber, and *R_f_* signifies the fiber radius.

Focusing on the mandrel’s radial vibration resonance, a Hooke’s law-type relation can be expressed. According to Equation (4), it follows that:

(9) $$K_{\mathrm{eff}}=\pi \frac{EHR-\mu E_{f}S_{f}N}{\mu R}$$

By substituting Equations (8) and (9) into Equation (7), the radial equivalent resonant frequency *f*_c_ is obtained. Inserting *f_c_* into Equation (6) yields the optical phase response.

The modeling analysis indicates that, with a fixed wound-fiber length, the frequency sensitivity of a mandrel-type fiber-optic sensor is governed primarily by the mandrel’s material and geometry. The structural analysis and design of the mandrel are discussed next.

(1) Mandrel material

Since the mandrel is bonded directly to the stator-bar surface, its material must satisfy insulation requirements. Subsequently, the acoustic impedance matching with the main insulation should be considered, where the acoustic impedance *Z* is defined as the product of the medium density *ρ* and the sound speed *c* (*Z* = *ρc*) [24].

An acoustic wave of incident intensity *I_i_* at an interface between impedances *Z*_1_ and *Z*_2_ yields reflected and transmitted intensities *I_r_* and *I_t_*, respectively. The intensity transmission coefficient at the interface *t* is given by:

(10) $$t=\frac{I_{t}}{I_{i}}=\frac{4Z_{1}Z_{2}}{(Z_{1}+Z_{2}{)}^{2}}$$

Three widely used, easily machinable insulating materials were selected for comparison, with their key properties summarized in Table 1.

Equation (10) gives the acoustic-impedance matching factors of 0.54 for plastic, 0.23 for rubber, and 0.98 for epoxy laminate. Therefore, an epoxy resin plate (epoxy laminate) is selected as the mandrel material.

(2) Mandrel dimensions

Given the stator bar’s face width and slot-exit geometry, the mandrel radius is limited to 35 mm and the height to 20 mm. The numerical model is applied to determine suitable mandrel dimensions. This study employs a bend-insensitive single-mode fiber conforming to ITU-T G.657.A2 [25].

**Figure 4:**
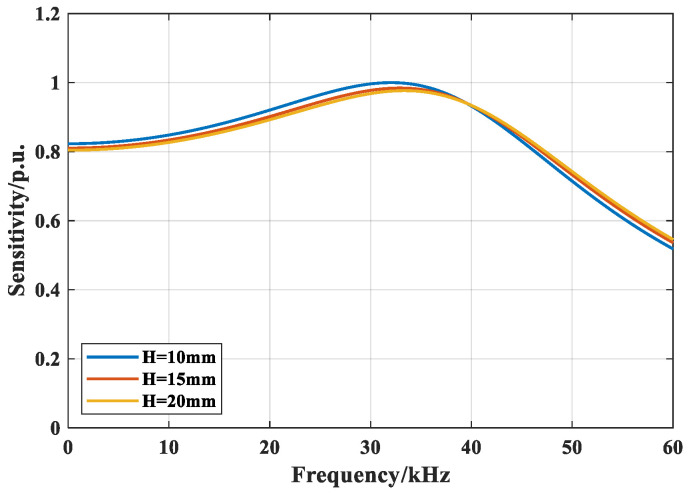
Sensitivity characteristics across varying mandrel heights.

The effect of mandrel height on sensor sensitivity is evaluated for a fixed radius of 20 mm. The frequency-domain sensitivities are computed for heights of 10, 15, and 20 mm, normalized to the maximum computed sensitivity (per-unit), as shown in Figure 4.

Figure 4 shows that there is minimal variation in the center frequency across the three heights. The 10 mm mandrel provides slightly higher sensitivity below 35 kHz, but the overall differences are marginal. At a fixed radius, increasing the mandrel height extends the fiber length per single winding layer and reduces the required number of layers, yielding a more uniform arrangement. Accordingly, a design height of 20 mm is adopted.

**Figure 5:**
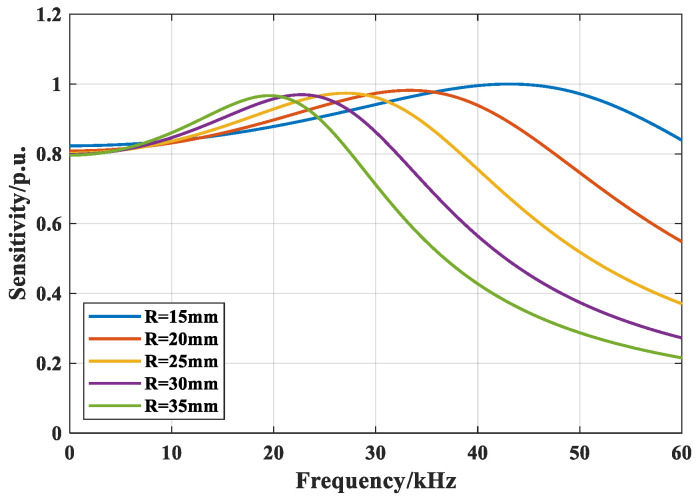
Sensitivity characteristics across varying mandrel radii.

With a fixed height of 20 mm, the frequency-domain sensitivity is computed for mandrel radii of 15, 20, 25, 30, and 35 mm, as shown in Figure 5.

Figure 5 indicates that increasing the mandrel radius shifts the center frequency downward, as a larger radius at fixed height increases the equivalent mass of the first-order (single-degree-of-freedom) vibratory system. The 15 mm radius yields the highest peak sensitivity, which decreases slightly with further radius enlargement. The sensitivity indices for the five radii are listed in Table 2.

Table 2 shows that the 15 mm radius exhibits the highest peak sensitivity but the lowest (15–30 kHz) average sensitivity. The 35 mm mandrel best matches the PD center frequency, though its average sensitivities over 15–30 kHz and 15–40 kHz are suboptimal. The 20 and 25 mm radii yield more balanced results. Prioritizing the 15–30 kHz average sensitivity, a 25 mm radius is adopted.

Theoretical analysis and simulations indicate that an epoxy laminate provides the most suitable acoustic impedance match to the main insulation compared with plastic and rubber. The mandrel height has minimal effect on frequency-domain sensitivity, whereas increasing the radius reduces the sensitivity. Considering the combined effects of material, height, and radius on sensor sensitivity, PD characteristics of stator bars, and practical installation constraints, the final design employs an epoxy cylindrical mandrel of 20 mm height and 25 mm radius, wound with two fiber layers totaling 20 m in length.

### 2.3. Sensor Performance Testing

(1) Sensitivity and linearity

**Figure 6:**
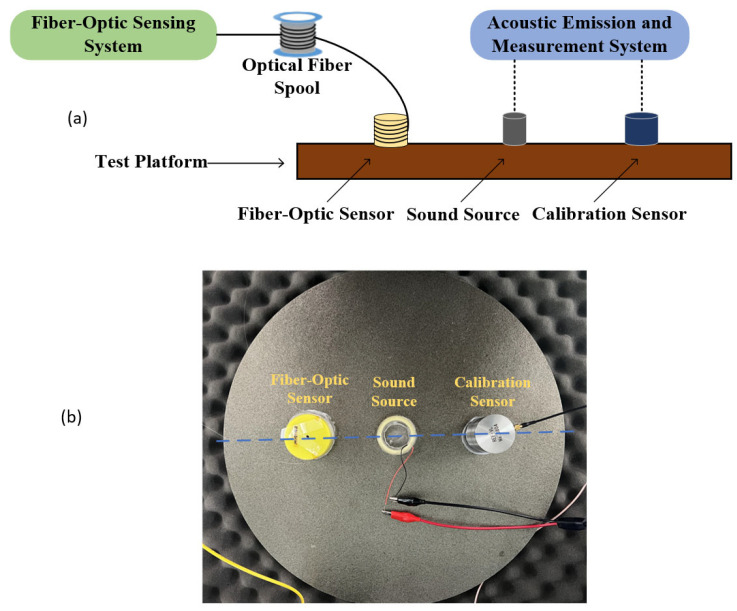
Testing platform for the fiber-acoustic sensor: (**a**) fiber-optic acoustic sensor test; (**b**) physical layout of the test rig. The blue dotted line indicates the alignment of the fiber-optic sensor, sound source, and calibration sensor.

With reference to relevant acoustic-emission sensor calibration standards [26], a fiber-optic acoustic sensor test platform was constructed, as shown in Figure 6.

The test block contains a 400 mm × 5 mm mica disk. A tubular PZT transducer served as the acoustic source, and a calibration-grade REF-VL sensor served as the reference. An acoustic couplant was applied at the sensor–platform interfaces. The reference sensor and the fiber-optic acoustic sensor were aligned with the acoustic source and positioned 100 mm from the disk center. Three fiber-optic sensors of identical external dimensions but varying fiber lengths, as listed in Table 3, were fabricated and flange-connected to a 2 km fiber spool. Measurements were repeated after each sensor replacement under identical conditions to ensure consistency.

**Figure 7:**
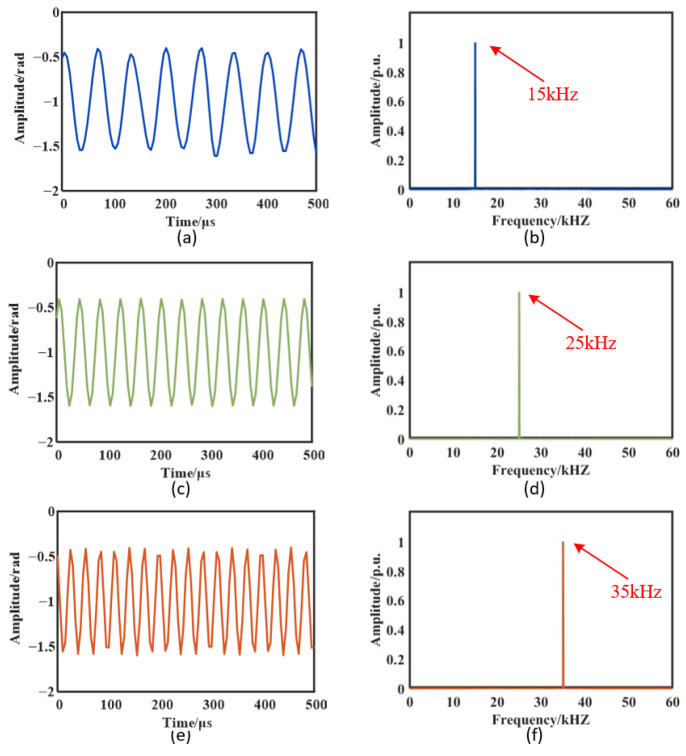
Detection performance at 15, 25, and 35 kHz: (**a**) 15 kHz time-domain waveform; (**b**) 15 kHz spectrum; (**c**) 25 kHz time-domain waveform; (**d**) 25 kHz spectrum; (**e**) 35 kHz time-domain waveform; (**f**) 35 kHz spectrum.

The PZT excitation was swept from 15 to 40 kHz in 1 kHz steps, with amplitude adjusted at each frequency. Synchronous measurements from the fiber-optic sensing system and the reference sensor were recorded. Figure 7 presents representative time-domain waveforms and spectra from Sensor 1 at 15, 25, and 35 kHz. The fiber-optic system reproduced the in-band sinusoidal excitation with high fidelity, yielding narrowband spectra free of harmonic distortion. These results demonstrate that the proposed RBS-based fiber-optic scheme provides an effective detection bandwidth without compromising range. Across the 15–40 kHz band, the system provides sufficient bandwidth margin to satisfy engineering requirements.

The frequency-domain sensitivities of the three fiber-optic sensors were calibrated from the preceding measurements. For comparison with the piezoelectric calibration sensor (voltage output, units V/(m·s^−1^)) and the fiber-optic sensor (phase output; units rad), 0 dB is defined as 1 V/(m·s^−1^). The corresponding fiber-optic sensor sensitivity is expressed as follows:

(11) $$S_{f}(f)=S_{0}(f)+20\log_{10}\frac{A_{f}(f)}{U_{0}(f)}$$

where *S*_0_(*f*) and *S_f_*(*f*) signify the sensitivity functions of the calibration sensor and the fiber-optic sensor, respectively, and *U*_0_(f) and *A_f_*(*f*) denote their measured values at each test frequency.

**Figure 8:**
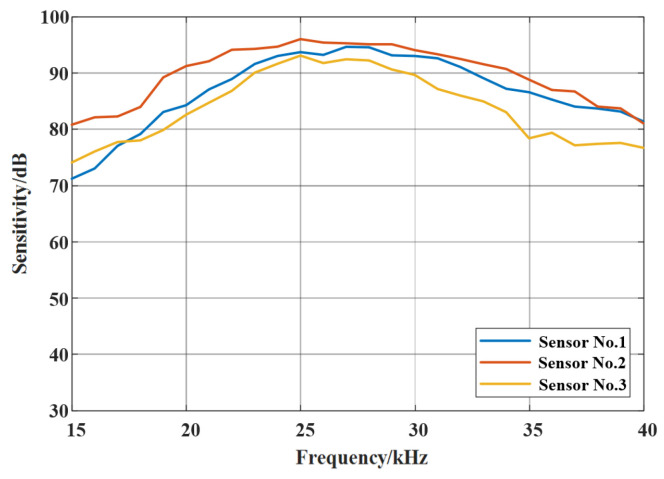
Detection performance of fiber-optic sensing systems across different frequencies.

The sensitivities of the three sensors were computed using Equation (11), with values derived from the root-mean-square (RMS) amplitudes of the measured waveforms, as shown in Figure 8.

Figure 8 illustrates that the center frequency of all the sensors, regardless of winding layer count, exhibits a center frequency near 25 kHz, as a small number of layers minimally alters the mandrel’s effective stiffness and mass.

Sensor 2 exhibits the highest sensitivity, while Sensor 3 demonstrates reduced performance. Therefore, extending the fiber length in mandrel-type sensors does not enhance detection, as additional layers suppress the mandrel’s dynamic response, and interlayer compression produces uneven loading, thereby offsetting the deformation benefits of the longer fiber.

**Figure 9:**
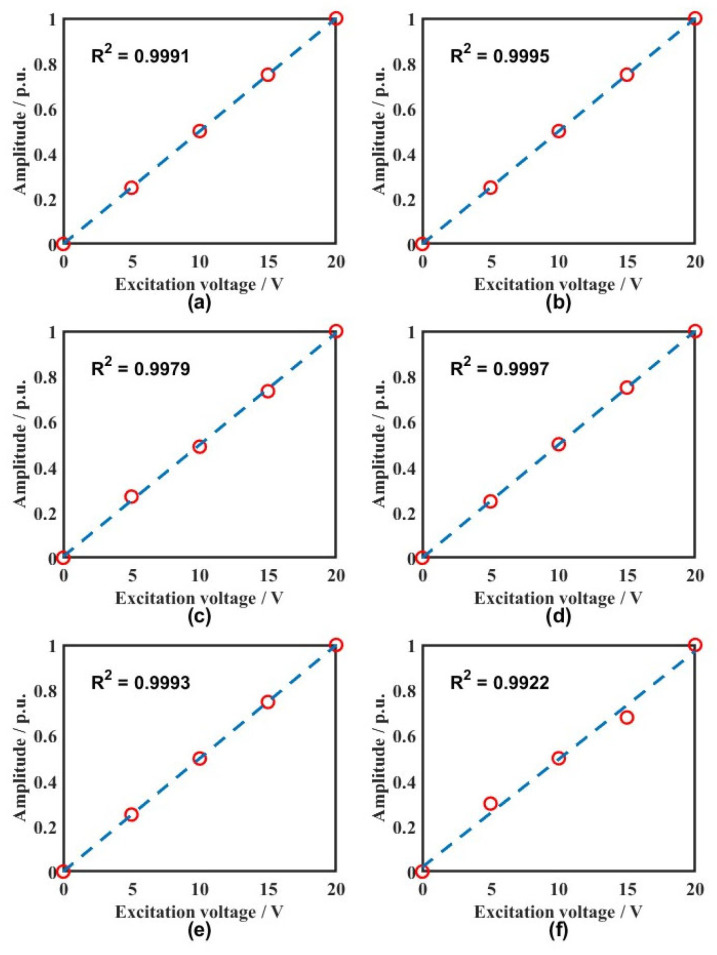
Detection linearity of the fiber-optic sensing system at 15–40 kHz: (**a**) 15 kHz; (**b**) 20 kHz; (**c**) 25 kHz; (**d**) 30 kHz; (**e**) 35 kHz; (**f**) 40 kHz.

Linearity was evaluated on the calibrated platform by sweeping the excitation from 15 to 40 kHz in 5 kHz increments, with source drives of 0, 5, 10, 15, and 20 V. At each frequency point, measurements were normalized to the fiber-sensor output at the maximum drive. Sensor 2, as shown in Figure 9, reveals that the fiber-optic sensor maintains a linearity above 0.99 across the tested band, reliably tracking signal amplitude variations with high system stability.

(2) Temperature effect test

**Figure 10:**
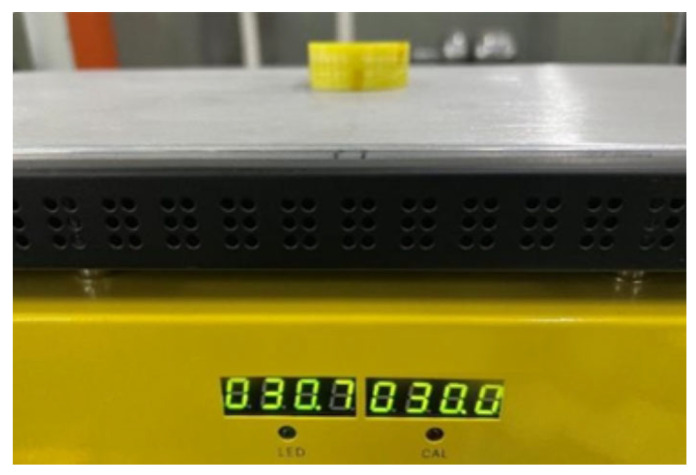
Fiber-optic sensor temperature testing platform.

During normal operation, the stator-bar surfaces may reach 85 °C [27]. Since the fiber-optic sensor is in direct contact with the stator-bar surface, the temperature effects must be considered. Given that thermal variation is much slower than PD acoustic signals, the temperature can be regarded as constant within a discharge cycle. Low-frequency thermal drift is mitigated by digital filtering, so temperature primarily contributes to measurement noise. In this study, steady-state thermal effects were examined using the heating platform, as shown in Figure 10, which maintained the platform’s temperature within ±1 °C.

The temperature was increased in 10 °C steps, and system noise was measured at setpoints between 30 °C and 100 °C. The noise level was quantified as the RMS of the noise waveform; the red dashed line indicates the measurement noise of the fiber-optic system at an ambient temperature of 17 °C.

**Figure 11:**
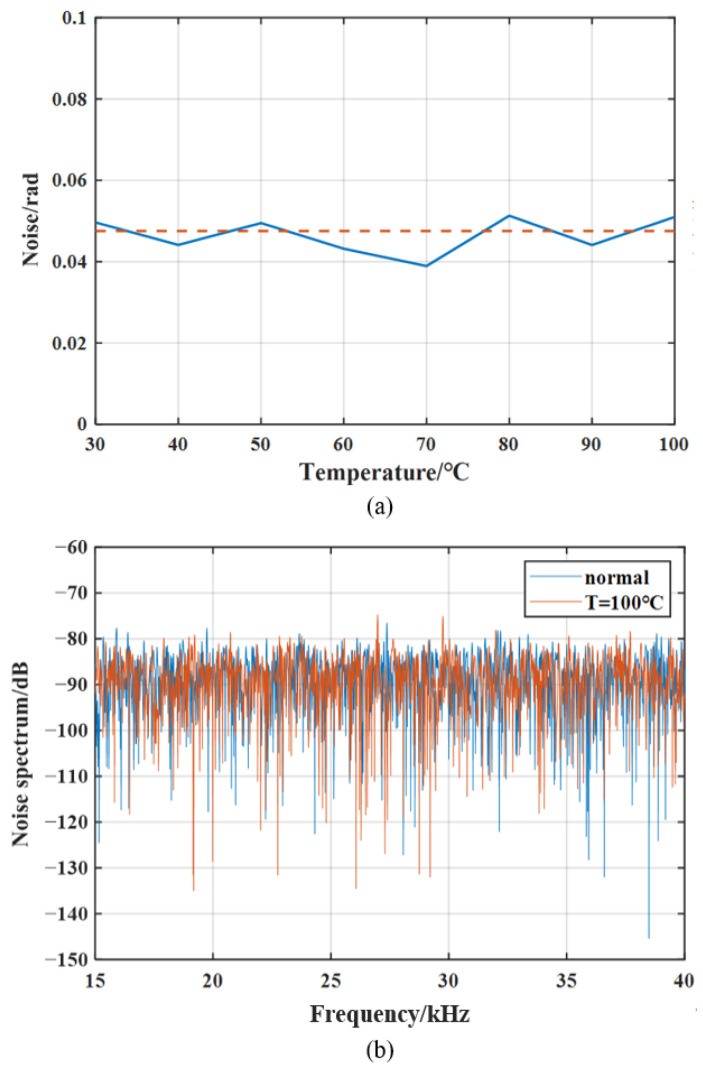
Temperature-induced noise of the fiber-optic sensor: (**a**) time-domain amplitude of temperature noise; (**b**) temperature noise spectrum. Colored solid lines show noise at different temperatures (30–100 °C), and the red dashed line indicates the measurement noise at 17 °C.

Figure 11 indicates that across the 30–100 °C range, the maximum measured noise is 0.039–0.051 rad with no obvious correlation between temperature and noise level. Therefore, temperature variations within the tested range do not increase measurement noise, confirming the fiber-optic sensor’s high system stability.

## 3. Stator Bar Partial Discharge Acoustic Test

### 3.1. Test Platform and Experimental Procedure

**Figure 12:**
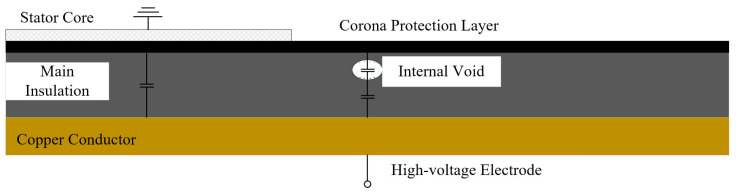
Schematic diagram of internal defects in the bar.

Partial discharges in stator bars typically originate from defects within the main insulation. The test object in this study was a full-scale stator bar. The length of the low-resistance straight section of the bar was 3 m, while the length of the high-resistance straight section after the corner transition was 0.5 m. The corner sections and end structures on both sides were symmetrical. Based on the characteristics of actual defects, an interlaminar insulation defect was artificially introduced inside the overlap region between the high- and low-resistance corona protection layers at the end of the straight section. The defect consists of three delaminated insulation layers with a gap of 0.3 mm. A schematic diagram of the internal defect in the stator bar is shown in Figure 12.

**Figure 13:**
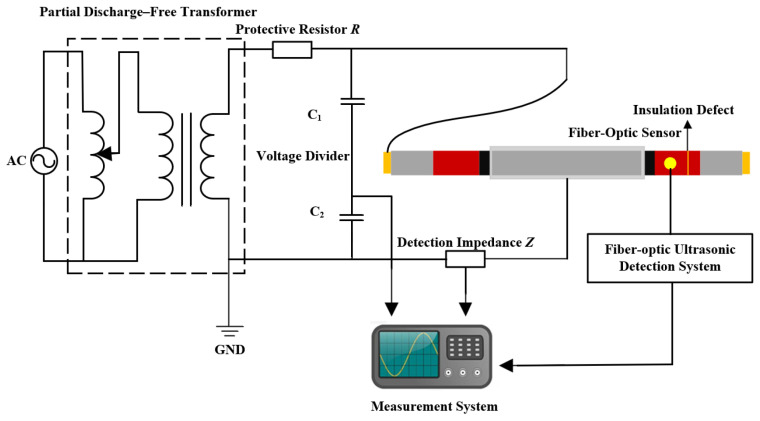
Partial-discharge optical fiber detection test platform for stator bar. The yellow region represents the copper conductor, the gray region the main insulation, the red region the high-resistance corona protection layer, and the black region the low-resistance corona protection layer.

**Figure 14:**
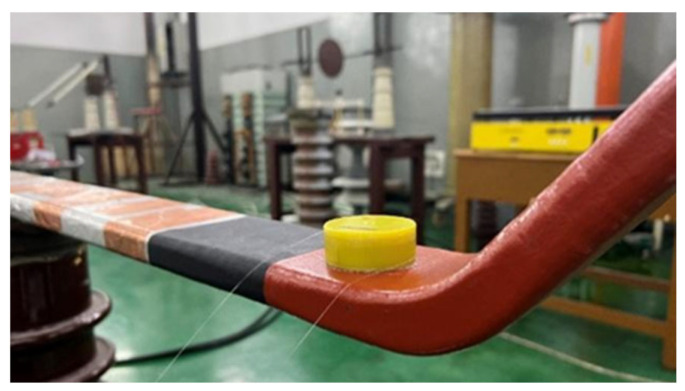
Layout of the fiber-optic sensor.

Figure 13 illustrates the fiber-optic PD detection platform for generator stator bars constructed based on the system described in Section 2. For subsequent data analysis, the apparent PD charge was obtained concurrently with the acoustic measurements using a detection impedance to capture pulse-current signals. A 20 m fiber-optic sensor was wound over the defect region, coated with couplant, and clamped tightly to ensure close contact. Since both the optical fiber and the mandrel are electrically insulating materials, installing the sensing fiber on the stator bar insulation does not significantly alter the electric field distribution on the stator bar surface and does not introduce additional partial-discharge risk. Figure 14 shows the installation configuration of the fiber-optic sensor.

### 3.2. Partial Discharge Magnitude Calibration

To ensure that the data collected by the pulse current method in the experiment can correctly reflect the partial discharge magnitude of the stator bar, the measurement circuit was calibrated before the voltage application test. The calibration method is in accordance with GB/T 7354-2018.

**Figure 15:**
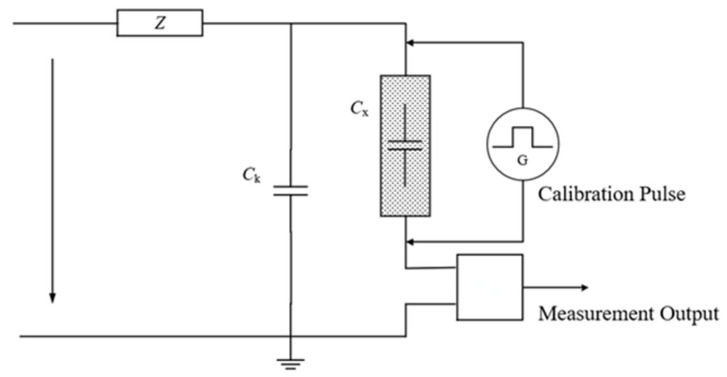
Partial discharge calibration test circuit.

**Figure 16:**
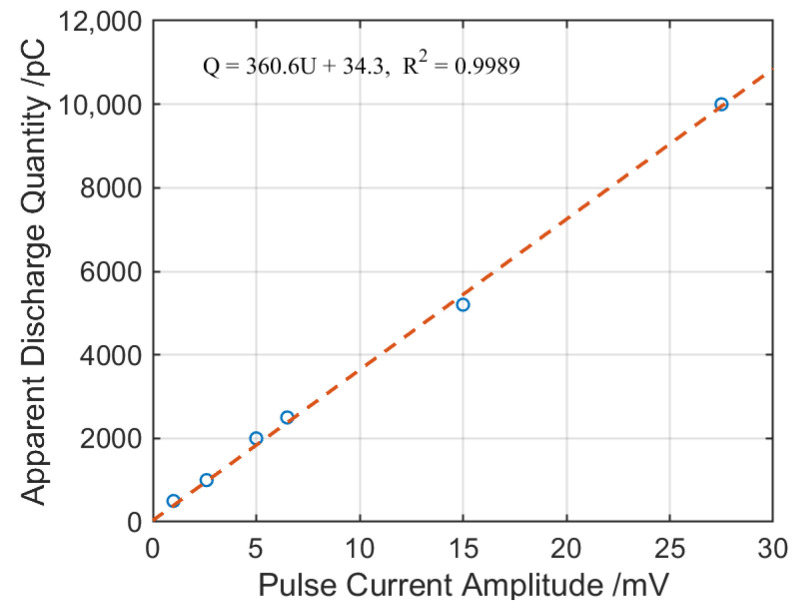
Discharge calibration curve.

As shown in Figure 15, *C_x_* and *C_k_* represent the test object capacitance and coupling capacitance, respectively, *Z* is the low-pass filter, and CD is the detection impedance. The pulse amplitude *U* was recorded under different injected charges *Q*, and the scale factor *k* of the apparent charge measurement was obtained through curve fitting, as shown in Figure 16.

The fitting equation is as follows:

(12) $$Q=360.6\cdot U+34.3,R^{2}=0.9989$$

**Figure 17:**
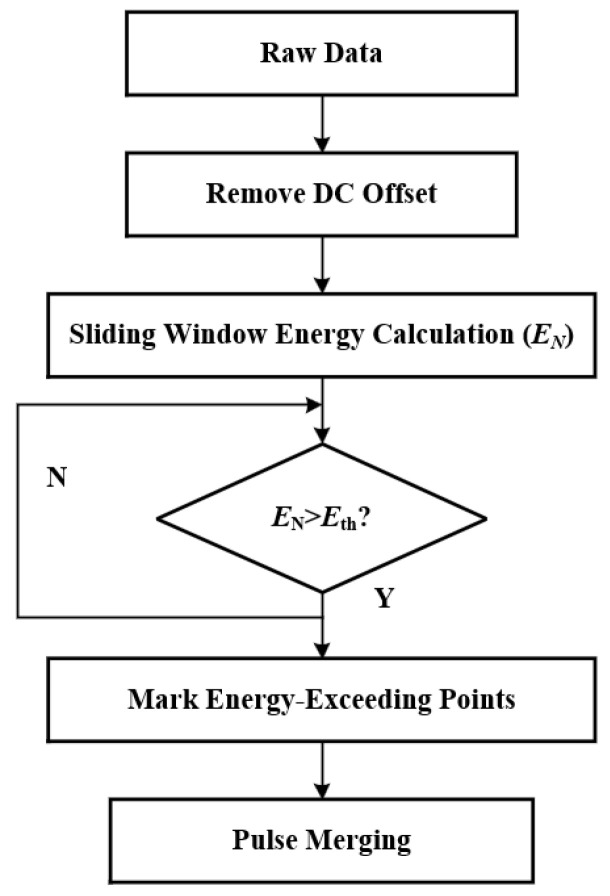
Algorithm for extracting discharge pulses.

The sliding-window energy detection method was adopted to extract discharge pulses. A sliding window *N* with a width slightly larger than the pulse width was defined, and the squared amplitudes of all signal samples within the window were accumulated to represent the total energy *E_N_* within the window. The discharge signal was scanned point by point with a step size of 1, and potential pulse intervals were identified by setting an energy threshold Eth to mark energy-exceeding points. The specific pulse extraction process is shown in Figure 17.

### 3.3. Test Results Analysis

**Figure 18:**
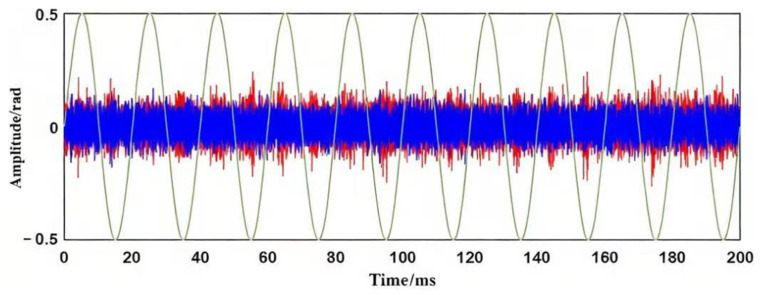
Discharge initiation signal detected by fiber-optic sensing system. The red line is the signal, the blue line is the noise, and green line represents the power-frequency voltage waveform.

At an applied voltage of 10 kV, the fiber-optic sensing system recorded signal bursts above the noise floor, corresponding to an apparent discharge of approximately 180 pC. Figure 18 shows the corresponding time-domain response.

**Figure 19:**
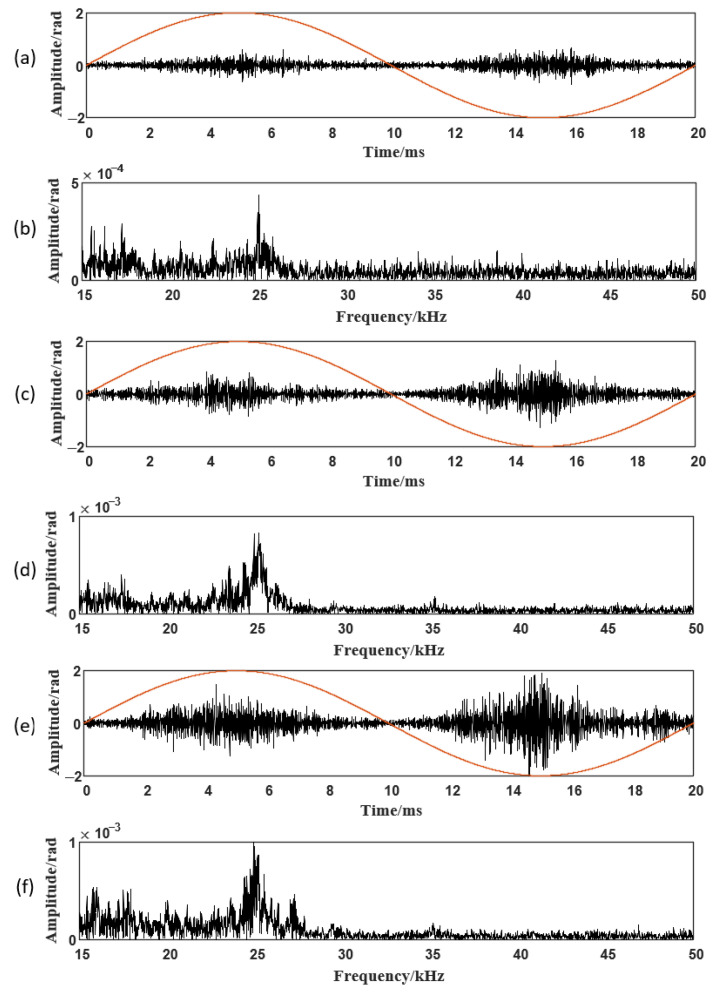
PD acoustic signals at 15, 20, and 25 kV: (**a**) 15 kV waveform. (**b**) 15 kV spectrum. (**c**) 20 kV waveform. (**d**) 20 kV spectrum. (**e**) 25 kV waveform. (**f**) 25 kV spectrum. The red lines represent the power-frequency voltage waveform.

Simultaneous measurements were obtained using the fiber-optic sensing system and the pulse-current method at various test voltages. Figure 19 shows the fiber-optic measurements over a single power-frequency cycle at 15, 20, and 25 kV.

**Figure 20:**
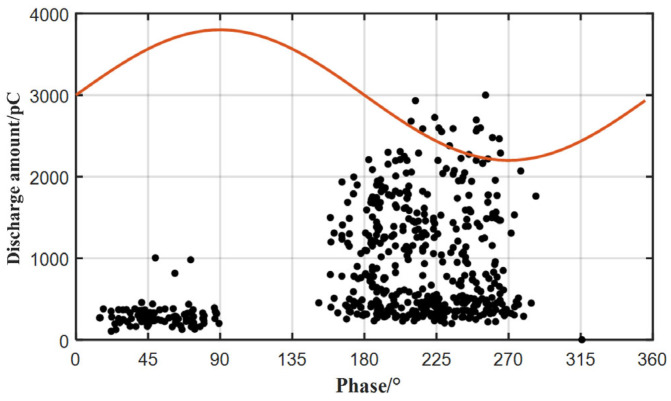
PRPD diagram of internal defects in the bar. The black dots represent the partial discharge events, while the red line represents the power-frequency voltage waveform.

Figure 20 shows the PRPD pattern of the internal defect in the stator bar generated from pulse-current data collected over 100 power-frequency cycles. The defect produces comparatively high discharge magnitudes, with stronger activity in the negative half-cycle. Discharges in the positive half-cycle are concentrated within the 0–90° voltage-rising interval, whereas discharges in the negative half-cycle are concentrated within the 135–270° interval.

From the fiber-optic measurements in Figure 19 and the PRPD pattern of the internal defect in Figure 20, the following conclusions can be drawn:

1. The fiber-optic sensing system accurately represents the discharge intensity, with higher applied voltages producing increased apparent charge, larger time-domain oscillations, and amplified spectral bands.

2. The measured signal energy is primarily confined to 15–30 kHz, with higher-frequency components partially unrecorded due to bandwidth limitations. The spectral peak near 25 kHz coincides with the designed peak sensitivity of the fiber-optic sensor.

3. The PD acoustic signatures of the stator bar exhibit pronounced asymmetry, with stronger activity in the negative half-cycle, consistent with the PRPD pattern of the internal defect. The acoustic signal exhibits a clear phase lag relative to the electrical PD signal.

In this study, an experiment was conducted on a stator bar with an artificial internal defect to investigate the acoustic characteristics of partial discharge signals. In practical generator monitoring, multiple fiber-optic sensors can be installed at the slot-exit positions of stator bars to achieve multi-point monitoring of partial-discharge acoustic signals in the stator winding.

## 4. Discharge Evaluation Method

### 4.1. Transformer–CNN–LSTM Hybrid Network Algorithm

At power-frequency voltage, partial discharge may occur in each cycle of the stator winding. Although a single discharge is a transient process at the ns scale, the acoustic signal excited by the discharge decays slowly. The instantaneous acoustic pressure amplitude is influenced by preceding discharges and thus exhibits strong temporal correlation. Therefore, within a single power-frequency cycle, there exists a strong temporal correlation among the acoustic signal data points collected by the fiber-optic sensing system.

**Figure 21:**
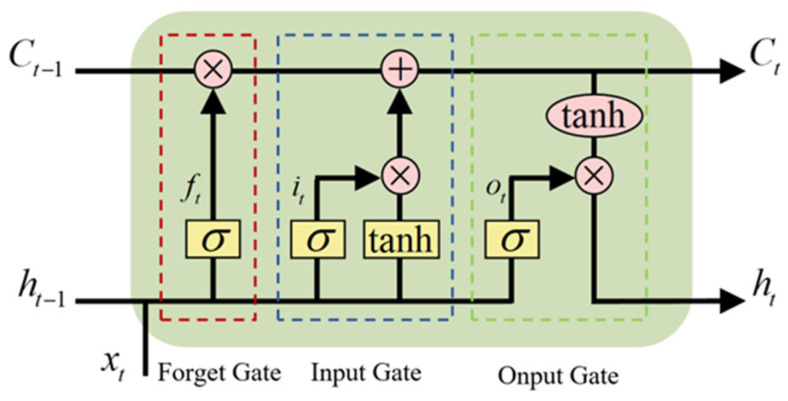
The basic framework of LSTM.

Recurrent neural networks (RNNs) enable networks to have memory functions through hidden nodes and are suitable for processing time series [28]. During model training using the back-propagation through time algorithm, problems with gradient explosion or vanishing made it impossible to obtain sequence features over long intervals [29]. Therefore, a gated structure, namely Long Short-Term Memory (LSTM) [30], was introduced into the RNN, as shown in Figure 21.

The gated structure consists of the forget gate, input gate, and output gate. The final output of the LSTM is jointly determined by the cell state and the output gate, as follows:

(13) $${\tilde{C}}_{t}=\tanh(W_{c}\cdot [h_{t-1},x_{t}]+b_{c})$$

(14) $$c_{t}=f_{t}⊗c_{t-1}+i_{t}⊗C_{t}$$

(15) $$h_{t}=o_{t}⊗\tan h(c_{t})$$

where *C_t_* is the input cell state, and *W_c_* and *b_c_* represent the weight matrix and bias of the input cell, respectively.

Considering the ability of CNNs to extract local features, a CNN layer was inserted before the LSTM layer to perform local feature extraction and sequence compression, thereby improving modeling efficiency.

**Figure 22:**
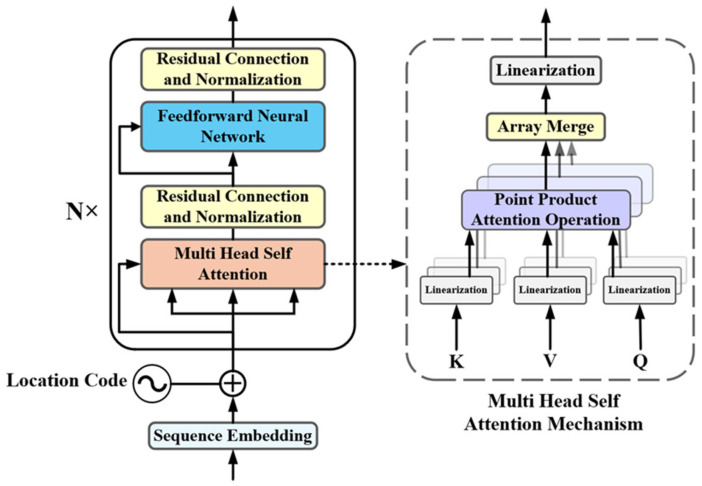
The structure of the Transformer encoder.

For partial discharge inside a stator bar, the superposition of acoustic signals depends on the occurrence time of discharge pulses. In the pulse current method, discharge pulses can be directly observed or extracted using threshold gating. In contrast, acoustic signals lack distinct single-pulse characteristics, and the occurrence time is embedded within the continuous variation in amplitude. Therefore, the multi-head self-attention mechanism of the Transformer encoder [31] was introduced into the model for further optimization. The encoder structure is shown in Figure 22.

The Transformer encoder projects the input into an embedding space through a fully connected layer, and the multi-head self-attention mechanism further partitions the input into multiple subspaces, where the Query, Key, and Value vectors are computed using the corresponding projection matrices:

(16) $$\left[Q,K,V]=X\cdot [W_{Q},W_{K},W_{V}\right]$$

The attention score of each head is computed according to the scaled dot-product attention formulation, and the outputs of all heads are concatenated. The parallel computation method enhances the representational capacity of the model and improves computational efficiency.

(17) $$\mathrm{Attention}(Q,K,V)=\mathrm{softmax}(\frac{QK^{T}}{\sqrt{d_{k}}})V$$

A feedforward neural network is subsequently applied to perform a nonlinear transformation on the attention output, and residual connections and layer normalization are employed to stabilize the training process and accelerate convergence.

In neural networks, regression analysis typically requires large data volumes and well-distributed label values, whereas the discharge magnitude values of internal defects in stator bars are relatively high and exhibit abrupt variations, which may reduce the reliability of regression modeling. Therefore, interval estimation of the average discharge magnitude within a specified time window was performed. All data were divided into *C* discharge-magnitude intervals, and the Softmax function was applied to convert the original output of the mode $z=[z_{1},z_{2},...,z_{C}]$ into a probability distribution:

(18) $${\hat{y}}_{i}=\frac{e^{z_{i}}}{\sum_{j=1}^{C}e^{z_{j}}},\;\;\;\;\;i=1,2,...,C$$

**Figure 23:**
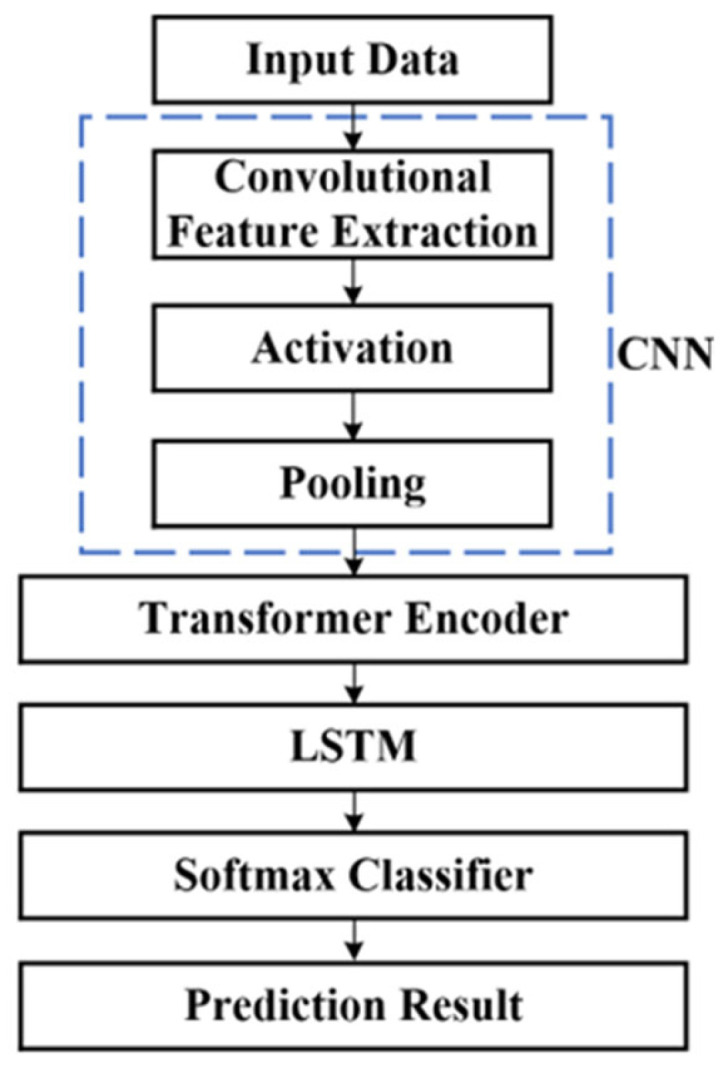
The overall architecture of a hybrid network.

The overall architecture of the hybrid network is shown in Figure 23.

By combining a multilayer CNN, a Transformer encoder, and an LSTM, the hybrid model can fully utilize the advantages of these three structures, thereby exhibiting stronger feature extraction ability and generalization ability when processing one-dimensional time-series data.

### 4.2. Evaluation of Partial Discharge Levels in Stator Bars

(1) Model Evaluation Metrics

In the constructed model, the classification output is a probability distribution processed by the Softmax function. For a classification task with *C* categories, the output for a sample can be expressed as follows:

(19) $$\hat{y}=[{\hat{y}}_{1},{\hat{y}}_{2},...,{\hat{y}}_{C}]$$

where ${\hat{y}}_{i}$ is the probability predicted by the model that it belongs to the *i*-th category.

The cross-entropy loss function is used as the optimization objective during model training. When the dataset labels are encoded using one-hot representation, the loss function can be defined as follows:

(20) $$Loss=-\log({\hat{y}}_{t})$$

where ${\hat{y}}_{t}$ is the probability corresponding to the true label in the model prediction.

In binary classification problems, TP (true positive) denotes the number of samples correctly predicted as positive by the model, TN (true negative) denotes the number of samples correctly predicted as negative by the model, FP (false positive) denotes the number of samples incorrectly predicted as positive by the model, and FN (false negative) denotes the number of samples incorrectly predicted as negative by the model. Commonly used model evaluation metrics are shown in Table 4.

For the partial discharge classification task, the one-versus-rest strategy was adopted, in which samples from one category are labeled as positive and samples from the other categories are labeled as negative. The corresponding metrics were first calculated for each category and then aggregated using the macro-averaging method.

(2) Dataset Partitioning and Parameter Settings

The acoustic signal measurement data from the fiber-optic sensing system were recorded at different test voltages, and the aggregated experimental data were segmented. Each input sample corresponds to a data segment extracted from the continuously acquired acoustic signals of the fiber-optic sensing system, covering five power-frequency cycles.

The discharge-magnitude intervals were defined, and all samples were classified accordingly. The number of samples in each discharge-magnitude interval (Labels 0–6) is summarized in Table 5. In each category, the data were divided into a training set and a test set at a ratio of 8:2. The model was trained using the training dataset, while the evaluation metrics were calculated based on predictions from an independent hold-out test set.

To improve model generalization and mitigate overfitting, the model parameters were set as follows. The CNN component adopted a three-layer convolutional architecture, and a dropout layer with a rate of 0.2 was introduced after each CNN layer to prevent overfitting. In the Transformer encoder, a dropout layer with a rate of 0.2 was applied after the feedforward network, and a dropout layer with a rate of 0.5 was added after the LSTM layer. Other specific model settings are summarized in Table 6.

Based on the parameter settings listed in Table 6, the number of trainable parameters for the CNN, CNN-LSTM, and Transformer–CNN–LSTM models is approximately 3.6 × 10^4^, 1.6 × 10^5^, and 1.7 × 10^5^, respectively. Although the hybrid model introduces additional parameters, the overall model size remains relatively small and computationally manageable for practical industrial applications.

(3) Model Training and Evaluation

**Figure 24:**
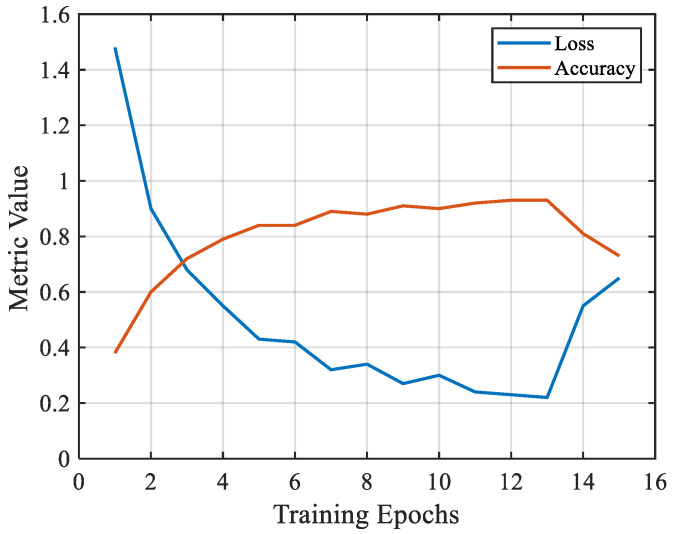
Changes in indicators during the training process.

The batch size for each training session was set to 32. The model traversed all samples within one training epoch, and each epoch contained 232 iterations. The loss and accuracy of the model after each training epoch are shown in Figure 24.

**Figure 25:**
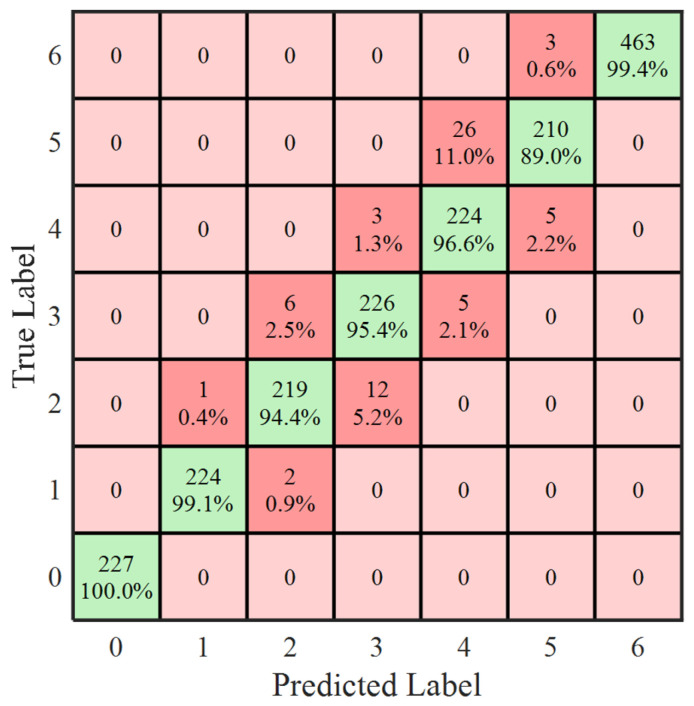
Confusion matrix of the test set. Green indicates correct classifications and red indicates misclassifications; darker colors represent higher rates.

As shown in Figure 24, the convergence trend of the model was evident, and both indicators reached their optimum values after 13 training epochs, corresponding to 3016 iterations. At this stage, the training accuracy reached 92.5%, and the loss decreased to 0.22. The classification performance of the model is illustrated in the confusion matrix in Figure 25, and the corresponding performance metrics (Accuracy, Precision, Recall, and F1-score) are listed in Table 7, which were calculated based on predictions from an independent hold-out test set.

The model achieved the highest classification accuracy in the low-discharge-magnitude interval (below 100 pC, label 0), reaching 100%, indicating that false detections would not occur when the partial discharge magnitude was low and thus would not interfere with operational decision-making. The classification accuracy in the high-discharge-magnitude interval (above 2000 pC, label 6) was the second highest, reaching 99.4%, demonstrating sufficient capability for timely assessment when the discharge magnitude approached the critical threshold. The overall recognition accuracy reached 96.6%. For all test samples, incorrectly estimated intervals were distributed adjacent to the correct interval, with numerical deviations less than 500 pC.

**Figure 26:**
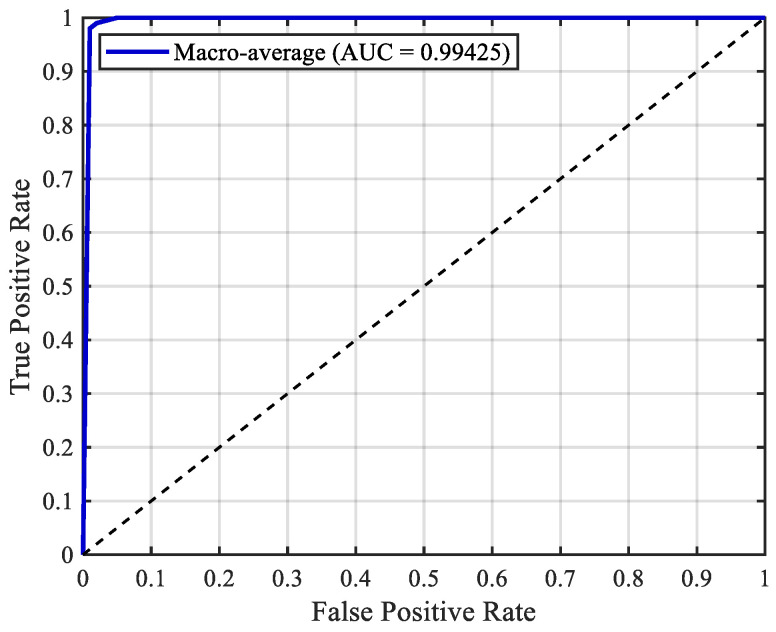
ROC curve of the test set. The dotted line represents the performance of a random classifier.

The receiver operating characteristic (ROC) curve was used to evaluate the performance of the classification model under different thresholds, and performance was quantified by the area under the curve (AUC). The one-versus-the-rest strategy was adopted to calculate the ROC curve for each category, followed by macro-averaging of the true positive rate and false positive rate across all categories. The results are shown in Figure 26.

The AUC value reached 0.99425, which was close to 1, indicating excellent performance in the multiclass classification task.

To further illustrate the advantages of the model constructed in Section 4.1, a CNN model and a CNN-LSTM model were also constructed using the same dataset for training. The comparison results are presented in Table 8.

The results indicate that the pure CNN model exhibited limited discrimination capability for discharge-magnitude intervals, achieving an accuracy of 73.1%, whereas the CNN-LSTM model, benefiting from temporal feature modeling, achieved a classification accuracy of 89.3%, which was significantly higher than that of the CNN model. After introducing the attention mechanism through the Transformer encoder, the classification accuracy of the hybrid model was further improved by 7.3% compared with CNN-LSTM. Moreover, the hybrid model required significantly fewer training epochs than the other two models.

## 5. Conclusions

This study presents a fiber-optic acoustic detection approach for monitoring PD in stator bars. A mandrel-type fiber-optic acoustic sensor was developed, and PD tests were conducted on full-scale stator bars with internal defects. Meanwhile, a hybrid neural network model integrating a CNN, an LSTM, and a Transformer encoder was proposed to perform interval estimation of the stator-bar partial discharge magnitude. The main conclusions are summarized as follows:

1. An RBS-based fiber-optic acoustic detection system was developed for stator-bar PD monitoring, featuring a mandrel-type fiber sensor with a height of 20 mm and a radius of 25 mm. Performance tests indicate a center frequency of 25 kHz, matching the dominant PD acoustic signals, with sensitivity consistent with theoretical modeling and linearity exceeding 0.99. The sensing system demonstrates high stability, remaining largely unaffected by temperature fluctuations within the range of 30–100 °C.

2. PD tests on a full-scale stator bar with internal defects confirm that the proposed fiber-optic acoustic detection system can detect weak time-domain acoustic signals corresponding to a discharge magnitude of approximately 180 pC, and the signal characteristics reflect discharge severity. The acoustic signal during the negative half-cycle of the power frequency is stronger than that during the positive half-cycle, consistent with the PRPD characteristics of internal defects, and exhibits a clear phase lag relative to the electrical signal.

3. The overall estimation accuracy of the Transformer–CNN–LSTM hybrid model for the partial discharge magnitude intervals of stator bars reached 96.6%, which was superior to the CNN (73.1%) and CNN-LSTM (89.3%) models and required fewer training epochs. The estimation accuracy of the hybrid model in the low-discharge magnitude interval (below 100 pC) and the high-discharge magnitude interval (above 2000 pC) reached 100% and 99.4%, respectively, with a low risk of misclassification.

Future work will focus on more detailed classifications of internal stator bar defects (e.g., insulation interlayer delamination and insulation–conductor delamination) and experiments under different operating conditions. Expanding the dataset of partial-discharge acoustic signals will further improve the evaluation capability and generalization performance of the proposed model.

## Figures and Tables

**Table 1:** Material properties of the mandrel.

Material	Density (kg/m^3^)	Young’s Modulus (Gpa)	Poisson’s Ratio
Main insulation	1173	26.3	0.32
Plastic	2100	0.6	0.42
Rubber	1500	0.1	0.47
Epoxy board	2000	25	0.38

**Table 2:** Center frequency simulated across different core axis radii.

Radius *R* (mm)	15	20	25	30	35
Center frequency	43.10	33.34	27.06	22.73	19.58
Peak sensitivity	1	0.9819	0.9738	0.9694	0.9668
15–30 kHz Average sensitivity	0.8945	0.9179	0.9415	0.9387	0.8978
15–30 kHz Average sensitivity	0.9252	0.9393	0.9147	0.8474	0.7607

**Table 3:** Various sensor parameters.

Sensor Number	Mandrel Size	Fiber Layer	Fiber Length
1	R25 × 20 mm	1	10 m
2	R25 × 20 mm	2	20.2 m
3	R25 × 20 mm	3	30.5 m

**Table 4:** Classification model evaluation indicators.

Metric	Definition
Accuracy	$\frac{TP+TN}{TP+TN+FP+FN}$
Precision	$\frac{TP}{TP+FP}$
Recall	$\frac{TP}{TP+FN}$
F1 Score	$2\times \frac{\mathrm{Precision}\times \mathrm{Recall}}{\mathrm{Precision}+\mathrm{Recall}}$

**Table 5:** Classification of the dataset.

Class Label	Risk Level	Discharge Quantity Range (pC)	Number of Samples
0	Low	<100	1134
1		100~300	1130
2		300~500	1159
3	Medium	500~1000	1186
4		1000~1500	1162
5	High	1500~2000	1179
6		>2000	2327

**Table 6:** Parameter settings for the model.

Module	Parameter Name	Setting
CNN (Number of Filters × Kernel Size)	1st CNN Layer	32 × 7
	2nd CNN Layer	64 × 5
	3rd CNN Layer	128 × 3
Transformer	Embedding Dimension	64
	Number of Attention Heads	4
	Feedforward Network Dimension	128
LSTM	Number of LSTM Units	128

**Table 7:** Various indicators of the model.

Metric	Accuracy	Precision	Recall	F1 Score
Value	0.9661	0.9672	0.9661	0.9662

**Table 8:** Test results of different models.

Model	Training Epochs	Training Loss	Test Accuracy
CNN	35	1.052	73.1%
CNN-LSTM	21	0.629	89.3%
Transformer–CNN–LSTM	10	0.101	96.6%

## Data Availability

The data presented in this study are available on request from the corresponding author.
